# Visual perception of longitudinal waves: theory and observations

**DOI:** 10.1038/s41598-026-36204-y

**Published:** 2026-03-23

**Authors:** Christopher W. Tyler, Joshua A. Solomon, Stuart M. Anstis

**Affiliations:** 1 Smith-Kettlewell Eye Research Institute, San Francisco, USA; 2 City St George’s, University of London, London, UK; 3 University of California, San Diego, USA

**Keywords:** Neuroscience, Physics

## Abstract

We introduce the study of the visual perception of longitudinal travelling wave motion, which as a physical phenomenon forms the basis of acoustics and some forms of seismic transmission. A theoretical analysis of physical longitudinal wave motion reveals that it exhibits profound nonlinearities that have been almost entirely neglected by the physics community. We simulated longitudinal motion in visual form with a random-dot field in which each dot particle oscillates sinusoidally about a fixed position at the same frequency but with a phase advance proportional to its distance from the origin. The resultant longitudinal density wave is essentially sinusoidal at very low oscillation amplitudes, becoming progressively distorted as oscillation amplitude increases. Perceptually, the motion splits into a combination of forward motion of the crests and backward for the troughs, rather than a uniform travelling wave. When the maximum velocity of each dot particle equals that of the propagation, the density function approximates a narrow spike, which splits into a double spike at even greater amplitudes. Adding a single (‘rigid’) velocity component can eliminate either the forward or backward percept. Remarkably, the speed needed for perceptual cancellation scales with oscillation amplitude, but nonlinearly so for the forward crest motion. Longitudinal waves evoked no motion aftereffect at any amplitude unless the contrast of the forward crest motion was reduced, revealing a motion aftereffect from the now-dominant retrograde trough motion. These unexpected results underline the emergent, or higher-order, nature of the perception of longitudinal travelling wave motion.

**Supplementary Information:**

The online version contains supplementary material available at 10.1038/s41598-026-36204-y.

## Introduction

**Fig. 1:**
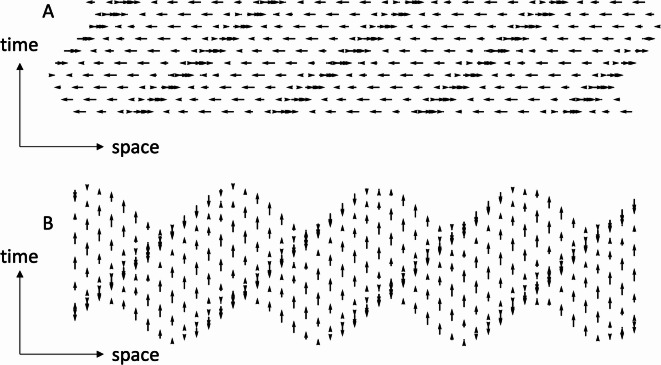
Static illustrations of (**a**) longitudinal and (**b**) transverse waves, propagating rightward through a grid of dots. (See Supplementary Movie 1 for a dynamic version of this figure.) For illustrative purposes, equilibrium positions of the dots are equally spaced. In all subsequent movies, equilibrium positions were selected at random from a uniform distribution.) In each panel, 10 successive temporal samples of the wave have been arranged vertically. Each dot has been replaced by an arrow indicating the direction and speed of its motion.

Any travelling wave can be decomposed into longitudinal and transverse oscillations. In mechanical waves, those oscillations are applied to the positions of particles within the solid, liquid, and/or gaseous medium through which the wave travels. (Electromagnetic waves, on the other hand, can travel through a vacuum.) Sound waves in gases are wholly longitudinal. Particles are displaced away from and back toward the origin of the sound. These oscillations can be illustrated with a matrix of horizontally moving dots, as in Supplementary Movie 1a (diagrammed in Fig. 1a). Transverse waves can also be illustrated with a matrix of moving dots (Supplementary Movie 1b; diagrammed in Fig. 1b). Note that the longitudinal wave and the transverse wave depicted in Supplementary Movie 1 in both propagate rightward, but whereas each individual dot in Supplementary Movie 1a oscillates horizontally, each individual dot in Supplementary Movie 1b oscillates vertically. In both cases, what is propagating is not the local elements per se but the phase of the oscillations, which advances from left to right.

Whereas some perceptual qualities of transverse waves travelling through visual texture^1,2^ have been studied using psychophysical paradigms like those used with drifting luminance (e.g.^3^) and chromatic (e.g.^4^) gratings, our focus here is on the properties of longitudinal waves, in which local oscillations are parallel to the wave propagation.

### Longitudinal wave motion

Analysis of physical longitudinal wave motion reveals that it exhibits profound nonlinearities that are largely neglected in the physics community. Longitudinal motion is almost universally treated with the small amplitude approximation, where a sinusoidal driving function is assumed to generate an essentially sinusoidal travelling wave, in the longitudinal wave case just as in the transverse wave case. Our visualizations of the molecular behavior of the sound wave in a gaseous medium clarified the extent of the physical deviations from this approximation and led to a series of explorations of the unexpected perceptual properties of the visualized. 

Historically, a form of high amplitude nonlinearity for pressure shock waves was recognized early in the C19th by Poisson^5^ and elaborated by Challis^6^ and Stokes^7^. Visualizations of longitudinal wave motion proliferate in textbooks on sound waves and on the internet. Particles comprising a longitudinal wave in an idealized gas are typically represented by dots, whose positions often oscillate sinusoidally, as though perturbed by a pure tone input. Such dynamic illustrations have undeniable value as teaching tools, but problems arise when they are accompanied by a graph of the density function. In every example we have found, this graph depicts the density is also sinusoidal. In actuality, the density of particles in a longitudinal wave is not sinusoidal but steepens as an accelerating function of the amplitude of the driving function (e.g.^8^) to produce the narrow peaks and broad troughs that are evident in the travelling-wave simulations. We first provide the theoretical analysis of these ‘ideal’ nonlinearities and then explore their perceptual effects when the longitudinal waves are rendered as dynamic visual displays.

## Formal specification

Following Zeleny et al.^9^, we assume that each particle in a longitudinal wave oscillates around a fixed position ${x}_{0}$, called its equilibrium position. At any time $t$, its position $x$ can be described as

1 $$x = x_{0} + a {\mathrm{sin}}\left[ {\omega \left( {\frac{{x_{0} }}{c} - t} \right)} \right],$$

where $a$ is the amplitude, $\omega$ is the angular frequency, and $c$ is the propagation speed. (Note that longitudinal waves in a 3D medium propagate three-dimensionally (though anisotropically) from the (1D) source of vibration, whereas transverse waves can only propagate two-dimensionally in a 3D world. For convenience, we will assume that the fixed positions are randomly sampled from a 1D uniform distribution. Wavelength is defined as

2 $$\lambda = \frac{2\pi c}{\omega }.$$

**Fig. 2:**
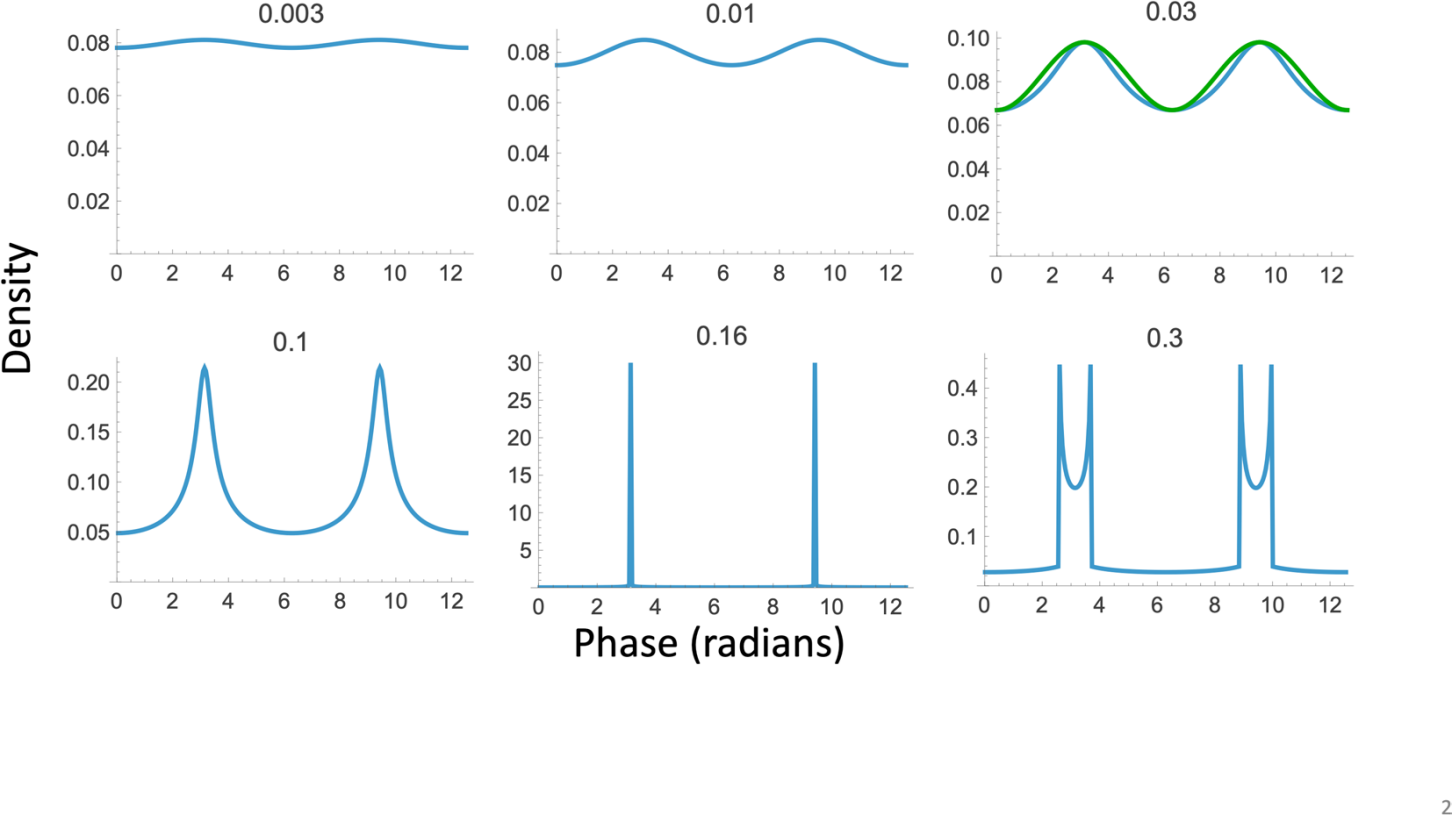
Longitudinal-wave density functions (blue curves) with wavelength $\lambda =2\pi$ and oscillation amplitudes ranging from $a=0.003\lambda$ to $a=0.3\lambda$. The green curve at $0.03\lambda$ is a true sinusoid whose peak-to-peak amplitude equals that of the density. Note that the waveform at higher amplitudes resembles the double-peaked form at $0.3\lambda$ but with wider spiked bars.

Each panel in Fig. 2 shows the density function of the travelling wave over space for $2$ wavelengths. There seems to be no closed-form solution for this function. Instead, we fixed $c=\omega ,$ $t=0,$ and used numerical methods (Mathematica’s NIntegrate) to compute the cumulative distribution function over $1$ wavelength of ${x}_{0}$ (normalized by its maximum value as specified in the denominator of the fractional term), differentiated with respect to $x$, and plotted the resulting density function, i.e.,

3 $$f_{X} \left( x \right) = \frac{d}{dx} \frac{{\mathop \smallint \nolimits_{0}^{\lambda } H\left[ {x - x_{0} - a {\mathrm{sin}}\left( {x_{0} } \right)} \right] dx_{0} }}{{\mathop \smallint \nolimits_{0}^{\lambda } H\left[ {\lambda - x_{0} - a {\mathrm{sin}}\left( {x_{0} } \right)} \right] dx_{0} }},$$

where $H$ denotes the Heaviside step function.

**Fig. 3:**
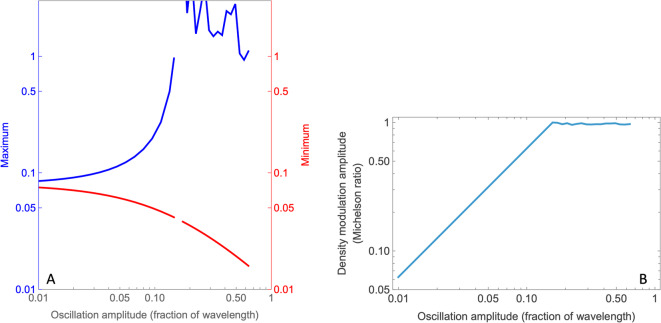
(**A**) Nonlinear relationship between oscillation amplitude ($a/\lambda$) and a density maxima and minima, plotted on double-logarithmic coordinates. (**B**) Travelling wave Michelson amplitude given by the expression $\left[\mathrm{max}{f}_{X}\left(x\right)-\mathrm{min}{f}_{X}\left(x\right)\right]/\left[\mathrm{max}{f}_{X}\left(x\right)+\mathrm{min}{f}_{X}\left(x\right)\right]$. The jagged appearance beyond amplitude of 0.15 is an artifact of the numerical methods used to compute these densities.

The behavior of Eq. 3 is that increasing the oscillation amplitude does not merely increase the amplitude of the density modulation, it also changes the shape of the modulation from quasi-sinusoidal at low amplitudes (e.g., $a=0.003\lambda$) to spiky at moderate amplitudes (e.g., $a=0.16\lambda$) to a waveform with two peaks per wavelength (e.g., when $a=0.3\lambda$), as seen in Fig. 2. The maximum and minimum values of these function vary as nonlinear functions of the oscillation amplitude (Fig. 3A). Nevertheless, using the Michelson ratio [(maximum − minimum)/(maximum + minimum)] to index the density modulation, we find that modulation increases linearly with oscillation amplitudes up to $0.16\lambda$, where it hits a nonlinearity at a Michelson ratio of 0.95 (see Fig. 3). From this point, the amplitude appears to drift down slightly, although this portion of the curve is not defined to high accuracy due to the sampling limitations of the numerical methods used for this assessment. We note that these nonlinear relationships between oscillation amplitude and the density and contrast measures seem largely absent from the literature on the physics of sound.

Note that the ratio between each particle’s maximum speed and the propagation speed of the medium is $2 \Pi a/\lambda$. Thus, an individual particle will briefly move more rapidly than the longitudinal wave that it forms whenever $a > \lambda/(2 \pi)$. We suggest that the resultant distortion can be considered the cyclic equivalent of shockwaves, such as the sonic boom created when a jet moves faster than the speed of sound.

From the viewpoint of the usual sinusoidal approximation to this nonlinear function, it is important to know the levels at which it is applicable. A distortion criterion of < 1% root mean square deviation is exceeded at the $0.003\lambda$ amplitude and the criterion of < 10% is exceeded at the $0.03\lambda$ amplitude, the latter being depicted in Fig. 2 for visual reference. For comparison, these levels correspond to acoustic vibrations of about 0.1 and 1 mm in amplitude, respectively, for sound at 10 kHz travelling in air, which are well within the range of high-volume audio loudspeakers.

## Methods

The stimuli are presented as movies throughout the text. Mathematica or MATLAB was used to create each movie. The code for each movie can be downloaded from the website http://www.staff.city.ac.uk/~solomon/LongitudinalWaves.zip.

The phase-advancing sinusoidal oscillations of the longitudinal wave motion were overlaid on a field of random-dot particles representing an ideal gas. While $a<\lambda /\left(2\pi \right)$, Eq. 3 places local maxima and minima at odd and even multiples of $\pi$ radians, respectively. When $a>\lambda /\left(2\pi \right)$, the peaks double and odd multiples of $\pi$ radians become local minima. In that case, local maxima were found using Mathematica’s FindMaximum routine, which necessarily fails at the singular point when $a=\lambda /\left(2\pi \right)$. For all other oscillation amplitudes, sampling density was as close to as possible to 1024 phases per wavelength, subject to the constraints that all local maxima and local minima should be sampled, and all samples would be equally spaced. Note that derivative *d/dx* in Eq. 3 can be written $\underset{h\to 0}{\mathrm{lim}}\left[r\left(x+h\right)-r\left(x\right) \right]/h$, where $r\left(x\right)$ is the ratio of that equation’s two integrals, expressed as a function of $x$. This derivative was approximated using $\left[r\left(x+h\right)-r\left(x\right)\right]/h$, with $h={10}^{-10}$.

## Results

### Unipolar dot travelling waves

#### Stimuli

Inspired by the on-line resource created by Zeleny et al.^9^, we created visual renditions of annular longitudinal waves from Eq. 1, using a substrate of 600 oscillating random-dot samples whose equilibrium positions were randomly sampled from a uniform distribution over an annular region of a 2:1 radial extent. Supplementary Movie 2 contains 6 annular wave motions of increasing amplitude corresponding to the 6 panels in Fig. 2. The amplitudes are specified as its proportion of a wavelength.

Whereas rectangular dot arrays with horizontal or vertical oscillations like those illustrated in Fig. 1 tend to encourage eye movements in the direction of propagation (or possibly in the opposite direction), we opted to make perceptual judgments with annular dot arrays, because fixation at the center of an annulus discourages eye-movement tracking in any particular direction, keeping the dot array rotating at a fixed retinal eccentricity.

#### Observations

1. With fixation at the annulus center to avoid foveal tracking, it is difficult to appreciate that each dot is merely oscillating around a stationary position in the annulus. This oscillation can, however, be verified by foveating any individual dot within the annular band.
2. With central fixation, wave propagation is immediately apparent as the clockwise rotation of the eight compression regions (“crests”) around each of the annuli with sufficient amplitude.
3. The rarefaction regions (“troughs”) between crests appear to cohere into a uniform texture that rotates backwards (here, counterclockwise), even though the phase propagation direction is clockwise. This reverse motion thus represents the intrusion of the local motion of the individual dots for half the phase of the local oscillations, though perceived as a coherent reverse motion of the half-phase patches.
4. At the intermediate amplitudes, most observers can discern a subtle impression of depth, in which the compression regions appear closer to the viewer than the rarefaction regions.
5. With focal attention, the relative salience of individual crests and troughs may fluctuate, but the opposing directions of motion they convey can be experienced simultaneously.
6. The perceived speeds of rotation of the crests and troughs appear to increase with oscillation amplitude. This percept is robust, but it is an illusion because each crest (and trough) requires exactly 8.53 s to complete a full revolution around the annulus center.

### Density-luminance reciprocity

#### Stimuli

The high-density peak regions of our black-dot stimuli necessarily have a lower average luminance than the low-density trough regions. To determine if and how the visual perception of longitudinal motion was contingent upon this “reciprocity” between density and average luminance^10^, we created stimuli in which dot luminance was proportional to the average density of dots in each phase of the longitudinal wave. This manipulation eliminates the (expected) luminance contrast between crests and troughs, virtually eliminating their ability to stimulate standard motion-energy mechanisms, including the Reichardt detector^11,12^.

#### Observations

1. With luminance equated, the crests still appear to rotate forwards (clockwise) and the troughs backwards (counterclockwise), as in the original version.
2. The wave motion is fully visible for levels of 0.03λ and above.
3. The depth impression is similar to that for the original version with uncompensated luminance modulation.

This luminance-balanced control makes clear that the percept of the bidirectional wave motion is undiminished from the level of 0.03λ and above, suggesting that it can be conveyed by something other than standard, luminance-based motion-energy mechanisms.

### Polarity-randomized dots

#### Stimuli

Randomly selecting the polarity (black or white) of each dot is guaranteed to reduce any contrast between the average luminances of crest and trough, consequently minimizing the contribution from standard, luminance-based motion-energy mechanisms to the wave-motion percept. Examples of these “drift-balanced” stimuli^13^ are provided in Supplementary Movie 4.

#### Observations

All the observations made with black-dot stimuli (discussed in the section on unipolar dot travelling waves) apply equally to the polarity-randomized stimuli. Evidently, luminance contrast is not required for the perception of longitudinal wave motion, or for the visual segregation of the clockwise-propagating crests from the troughs, which again appear to rotate in the counterclockwise direction.

### Density-contrast reciprocity

#### Stimuli

The high-density peaks of our polarity-randomized stimuli necessarily have a more contrast energy than the low-density troughs. To determine if and how the visual perception of longitudinal motion was contingent upon this “reciprocity” between density and contrast energy^14^, we created stimuli in which the absolute value of each dot’s Weber contrast was inversely proportional to the average density of dots in each phase of the longitudinal wave. This manipulation reduces the angular modulation of contrast energy around each annulus, consequently reducing its ability to stimulate the “2^nd^-order” motion system, putatively responsible for computing the direction of spatiotemporal amplitude modulations^15^.

#### Observations

1. Contrast balancing weakens both clockwise and counterclockwise apparent motions, but both motions remain visible at moderate oscillation amplitudes ($0.03\lambda -0.1\lambda$).
2. At high amplitudes ($>0.16\lambda$), clockwise propagation of the crests becomes very hard to see, and the counterclockwise rotation of the trough regions dominate perception.
3. Anticipating the section on the motion aftereffect, the panels in the bottom row produce a weak motion aftereffect if observed when they stop after rotating for a while.

Luminance gratings may appear to drift transparently when their spatial frequency contents are very different. The crests and troughs of some longitudinal waves, on the other hand, appear to drift transparently even when their spatial frequency contents are similar, such as when the oscillation amplitude is 0.01λ or 0.3λ. This suggests that qualitatively different computations underlie the two directions of apparent motion. The fact that transparency can survive both luminance and contrast balancing further suggests that spatiotemporal modulations of contrast energy alone do not adequately describe either computation. Note however, that the clockwise motion of the contrast-balanced crests does eventually disappear when the oscillation amplitude becomes sufficiently large. This too is unlikely to be a by-product of amplitude’s effect on the relative widths of crests and troughs, because similarly wide crests are obtained with oscillation amplitudes of 0.01λ, where both directions of motion are visible, and 0.3λ, where clockwise motion is not visible. Thus, the disappearance of the clockwise crest motion with survival of the counterclockwise trough motion disqualifies the possibility that the disappearance is for crests that are narrow relative to some spatial integration area, because the crests at 0.3λ are as wide as those perceived at 0.1λ. The implication is that the crests and troughs are processed independently, and the reduced crest contrast weakens it as a stimulus for the crest motion but does not affect the strength of the trough motion. The motion aftereffect resulting from the residual counterclockwise motion is considered in the Motion aftereffect section and the Discussion.

What about spatiotemporal modulations of dot density? Note that several lines of evidence (e.g.,^14,16,17^) suggest that texture density is computed locally, early in the hierarchy of visual computations.

### Density waves

#### Stimuli

One inescapable feature of longitudinal waves is the periodic pile-up of particles in the direction of propagation. We test its ability to convey propagation when other features of the longitudinal wave have been removed. Although our contrast-balanced stimuli (described in the section on density-contrast reciprocity) have spatiotemporal modulations of neither luminance nor contrast energy, they still contain spatiotemporal modulations of motion: the average motion of crest dots is clockwise, while the average motion of trough dots is counterclockwise. Next, we virtually eliminated this local motion without changing the overall structure of our annuli by randomly re-assigning the radial position of each dot within the annulus on each frame, thus removing the local oscillatory dot motion but retaining the density modulation waves. The version of this stimulus in Supplementary Movie 6. tests whether waves of dot density in the absence of coordinated local motions of the dots provides a motion cue when compensated for the ancillary contrast modulation.

#### Observations

1. Having disrupted each dot’s trajectory, counterclockwise motion remains at neither the local level nor the global level.
2. No dot moves clockwise either, but the relatively dense, low-contrast crests continue to rotate physically in a clockwise direction around the annulus. That motion is very hard to see, but it does seem possible to track the motion of any individual crest with local attention.
3. There is a lot of flicker due to resampling the radial location of each dot in the annular region, including content at high temporal frequencies.

We conclude that waves of pure dot density with nulling of the consequent contrast modulation are almost invisible. The slight residual motion is likely attributable to an imperfect match of the dot density and compensatory contrast modulation functions.

### Flickering particles

Did the high temporal frequency content in our density waves simply mask their density modulation, or was the local motion of individual dots (absent from our density waves in previous sections) important for the appearance of contrast-balanced longitudinal waves?

#### Stimuli

To assess the impact of flicker on the visibility of contrast-balanced longitudinal waves, we created a new version of Supplementary Movie 5 in which dot polarity was reassigned randomly on each frame. Note that this manipulation doesn’t necessarily produce the same amount of flicker inherent in the density waves, but it does produce equally high temporal frequencies.

#### Observations

1. Flicker eliminates any appearance of coherent motion from the low amplitude ($<0.1\lambda$) annuli.
2. Flicker all but eliminates the appearance of propagation from the high-amplitude ($>0.1\lambda$) stimuli, but individual crests can be seen to rotate clockwise with effort.
3. When the oscillation amplitude $a=0.1\lambda$, all 8 crests can be seen to rotate coherently, but the counterclockwise rotation of the troughs dominates (as it does with higher oscillation amplitudes).

The introduction of random polarity reassignment unquestionably served to mask the apparent propagation of some of the contrast-balanced stimuli. It is conceivable that the apparent propagation of all our contrast-balanced stimuli could have been masked with more flicker. Consequently, it is uncertain whether the spatiotemporal modulation of dot density is sufficient to convey the impression of propagation amongst longitudinal waves, or whether a contribution from each dot’s oscillating trajectory is required. A contribution from those oscillations seems to be required for the impression of coherent, retrograde motion from the troughs.

### Cancellation of wave motion by opposing directions of rigid motion

The question arises how the apparent motions of crests and troughs are related to the local motions of the dots throughout the waveform. Clearly, neither direction of apparent motion corresponds to the average dot motion, because each dot is merely oscillating in place. Its motion is neither forward (clockwise) nor backward (counterclockwise) on average. We addressed this question by adding a rigid rotation to the travelling wave stimuli to determine at what speed it would be perceived to cancel either the clockwise crest motion or the counterclockwise trough motion. The default hypothesis is that the apparent speed is controlled by the rate of phase propagation, which is held constant in the following cancellation tests. Another possibility is that the apparent forward and backward motions correspond to the dots’ fastest forward and backward motions (with velocities ${{ \pm 2\pi ac} \mathord{\left/ {\vphantom {{ \pm 2\pi ac} \lambda }} \right. \kern-0pt} \lambda }$), respectively. Alternatively, the apparent velocities could correspond to the dots’ average forward or backward motions, respectively (with velocities ${{ \pm 4ac} \mathord{\left/ {\vphantom {{ \pm 4ac} \lambda }} \right. \kern-0pt} \lambda }$). (Note that, here and subsequently, we use the term ‘velocity’ to specify angular velocity relative to the center of the annular stimuli.)

#### Stimuli

To test between these alternatives, stimuli were generated with a range of rigid motions added to all the dots in the annular longitudinal waves. Supplementary Movie 8A-C illustrates a consensus when the oscillation amplitude had the high amplitude $0.25\lambda$ (i.e., beyond the linear range of Fig. 3B) In this case we judged the crests to be static when a rigid counterclockwise motion having a speed equal to 105% of the wave’s propagation (i.e. $1.05c$) was added to each dot. The troughs were judged to be static when a rigid clockwise motion having a speed equal to 150% of the wave’s propagation was added to each dot. The longitudinal wave in the central annulus has no additional rigid motion.

**Fig. 4:**
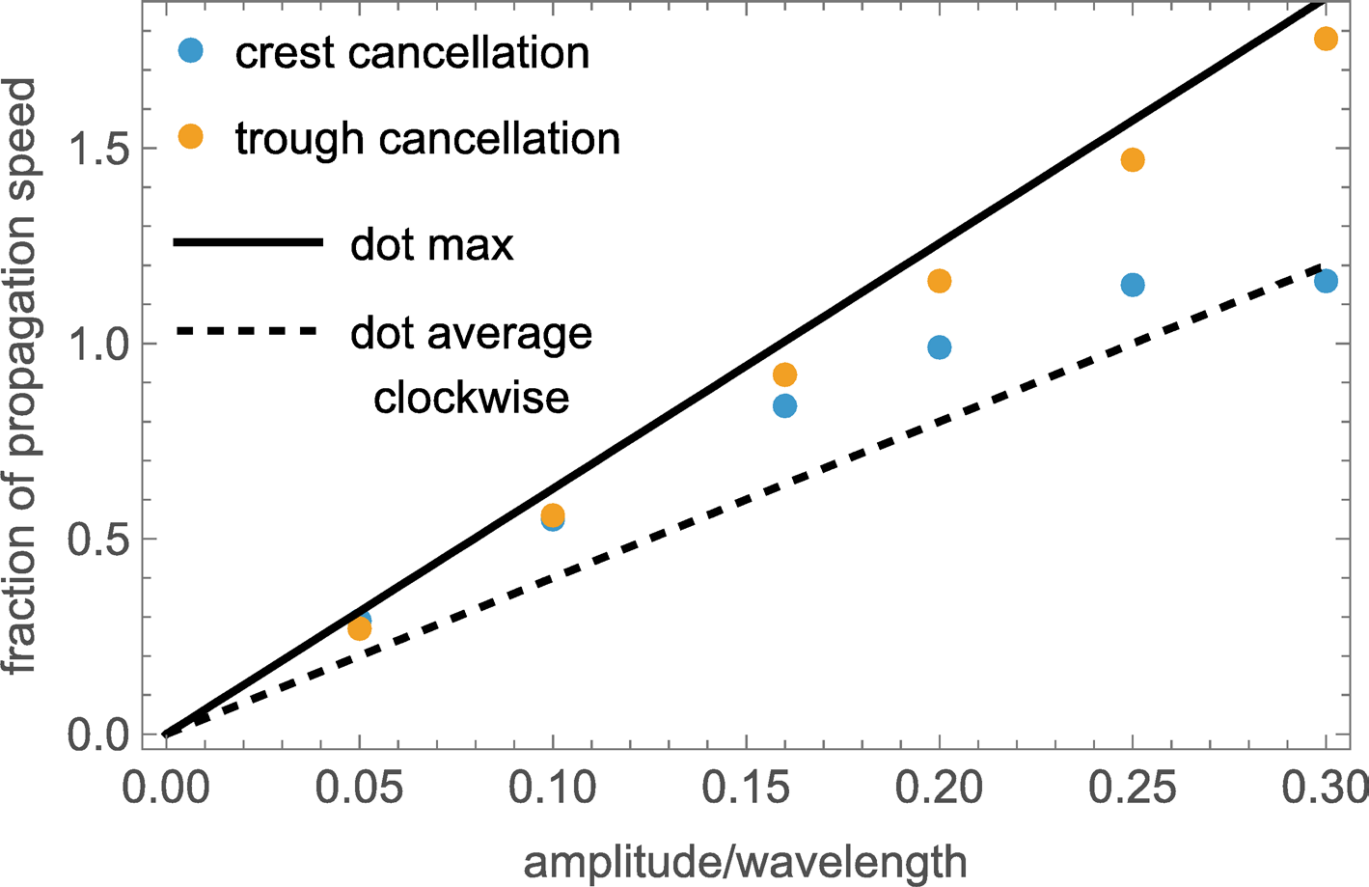
Cancellation speeds for the crest maxima and the trough minima of rotating longitudinal waves at a constant propagation speed of c = 42 deg/s. The only variable is the oscillation amplitude. The solid line plots the maximum dot speed without any additional rigid motion, while the dashed line plots their average speed. In each case, the physical clockwise and counterclockwise oscillation speeds of each dot are equal.

Panels 8D and F illustrate the same high-amplitude longitudinal wave with equal but opposite rigid velocities of +1.3*c*. This intermediate velocity proves too fast to cancel the crests’ motion (they appear to rotate counterclockwise in 8D) and too slow to cancel the troughs’ (they appear to rotate counterclockwise in 8F). Quantitative cancellation speeds for lower-velocity waves are plotted in Fig. 4.

 Supplementary Movie 9 contains the same longitudinal waves shown in Supplementary Movie 2, with the wave’s actual propagation velocity subtracted from each dot. This is approximately the correct speed for cancelling the apparent propagation of the crests only when the oscillation amplitude $a\approx 0.2\lambda$. For larger amplitudes, it is insufficient, and the crests appear to rotate clockwise. For smaller amplitudes, it is overkill, and any discernible crests appear to rotate counterclockwise.

These speed-cancellation observations reveal the remarkable result that the perceived propagation speed is some form of local average that lies between the particles’ average speed in that direction and their maximum speed. This is also true for the transparent, retrograde motion of the troughs, which seem to move a little faster. This difference in apparent speed increases with oscillation amplitudes > $0.1\lambda$, whereas the difference between the apparent widths of crest and trough is maximal when $a=0.16\lambda$.

#### Observations (see Fig. 4)

1. The cancellation speeds are not constant but scale monotonically with the oscillation amplitudes.
2. Setting the cancellation velocity to the average velocity of the crest or trough regions, respectively fails to cancel the perceived motion (except in one incidental case of the crest velocity at 0.3λ), invalidating the averaging hypothesis that the dot motion in whole of each half-cycle contributes to the perceived velocity. Effective cancellation requires added velocities closer to the maxima within each crest or trough.
3. For higher-amplitude waves, the trough-cancellation speed substantially exceeds the crest-cancellation speed.

These speed-cancellation observations reveal the remarkable result that the perceived wave motion depends on a property of the local dot motions whose oscillation is essentially invisible without guided scrutiny. This property is some form of local average that lies between their average speed and their maximum speed, separately and differently within the crest regions and trough regions$.$

### Motion aftereffect

### Stimuli

A question that does not seem to have been previously addressed in the perceptual literature is whether travelling wave motion generates a motion aftereffect. This may be viewed in a cyclic adaptation paradigm of 10-s adapting and 2-s test periods, as seen in Supplementary Movie 10. Note here that the high-amplitude longitudinal wave in panel B is identical to the one in Supplementary Movie 8B. The rotating texture in panel A has a velocity equal to the sum of the rigid velocities required to cancel the apparent motions of crests and troughs, as described in the previous section.

#### Observations

1. Even though the sum of (oppositely signed) velocities required to cancel the longitudinal wave’s opposite directions of apparent motion is small, that sum nonetheless elicits a strong motion aftereffect (Supplementary Movie 10A).
2. The longitudinal wave itself does not elicit any motion aftereffect (Supplementary Movie 10B).
3. Note also that the contrast-balanced wave of Supplementary Movie 5, which does not support the clockwise motion percept of the crests, does produce a weak motion aftereffect, which is stronger in peripheral vision.

These observations are consistent with evidence^18^ that the adaptation underlying the motion aftereffect occurs primarily in the directionally selective neurons of cortical area V1, where receptive fields are relatively small. Such neurons favoring clockwise motion would not be expected to receive any stronger stimulation than similarly positioned neurons favoring counterclockwise motion when individual dots are merely oscillating back and forth in their receptive fields. On the other hand, such neurons would receive stronger stimulation from rigid, clockwise motion. Consequently, the rigid motion should produce a significant aftereffect whereas the longitudinal motion should not. Indeed, this is what we found.

The motion aftereffect in the contrast-balanced conditions (Supplementary Movie 5) implies that the contrast-balanced reduction in the clockwise motion reduces the motion specific to the crest regions without affecting the counterclockwise trough motion, resulting in a counterclockwise bias that produces the clockwise motion aftereffect when the motion stops. This result in turn implies that the lack of motion aftereffect in the original stimulus is not because the local oscillations do not produce local motion aftereffects, but because these local aftereffects from the crest and trough motions cancel each other in the net effect.

## Discussion

Longitudinal waves are created from ensembles of locally oscillating dots with a progressive phase advance as a function of position. These oscillations create regions of compression and rarefaction whose propagation and retrograde motions so dominate perception that the local motion of individual dots is all but impossible to discern. These global, transparent motions thus arise by some process of motion integration.

We show that, even for locally linear transmission through the medium at constant speed, the density of the longitudinal wave motion becomes notably non-sinusoidal at oscillation amplitudes beyond about 2% of the wavelength, and progressively piles up into a narrow cyclic density spike around 16% of the wavelength, beyond which the peak splits into a double spike as the density accretion overtakes the maximum velocity of the local medium being perturbed. This nonlinearity is of entirely different character than the adiabatic shock wave nonlinearities that were the subject of contentious analyses in the C19th. Those turned out to be time-asymmetric in the direction of a sawtooth wave, whereas our analysis applies to speed-invariant Boylean gases, in which the density singularity for a sinusoidal input remains time-symmetric but becomes a double singularity at higher oscillation amplitudes.

Having established the full nature of longitudinal waves, we then turned to the perceptual appreciation of visual depictions of wave motion, such as are often used in acoustics courses. Instead of the usual linear wave of Fig. 1, however, we use rotating ring configurations of travelling wave motion to control eye movements. These show that the local dot motion in the wave is virtually invisible, but is subsumed under the global impression of motion, in which each cycle splits into two opposing regions: a dominant forward motion of the crest regions and a residual backward motion of the trough regions. This percept is obtained even when the oscillation amplitude is small enough that the wave motion remained sinusoidal. At higher amplitudes, the forward motion of the crests is enhanced by the nonlinear pile-up of the dot density there, which results in their being perceived as narrow ‘walls’ between broad ‘fields’ of opposing motion. In the rotating ring configuration, the individual cycles then integrated into transparent counterrotating motion fields. Perceptual binding of all 8 crests (or all 8 troughs), see Supplementary Movie 2 into a single coherent texture necessarily requires neurons having receptive fields large enough to be stimulated by them.

We then asked whether the appearance of the forward component of the longitudinal wave motion was attributable to the difference between the average luminances of the crests and troughs. This hypothesis was tested in two ways. First, we reduced the crests’ average luminance to match that of the troughs (Supplementary Movie 3). Second, we randomized dot polarity so that the expected luminance within each phase of the wave was equal to that of the mid-gray background (Supplementary Movie 4). Neither of these manipulations eliminated the impression of transparent, global rotations in opposite directions.

The impression of global motion in either direction can be eliminated by the addition of a uniform velocity to each particle. This cancelling velocity is close to the peak oscillation velocity (Supplementary Movie 8), but we have found that it varies with oscillation amplitude and direction (i.e., consistent with or opposite to that of propagation). While the cancellation of the trough motion was close, to proportional with the maximum trough velocity, the cancellation of the crest motion progressively declined below proportionality until it matched the average, rather than the maximum, of the crest velocity half-cycle of the waveform.

The distinction between a global property and the local components from which it is formed was highlighted by the Gestalt psychologists at the beginning of the twentieth century. The visual system’s ability to integrate local motion signals into a global percept has been recognized (e.g. by^19^) since the end of the twentieth century. Just as the Smith group sought to eliminate spatial frequency components from their random-dot stimulus that could potentially excite motion-energy detectors on a global scale, we too examined various “drift-balanced”^13^ modifications of our black-dot stimuli that were designed to hide their emergent properties from “1^st^-order” motion detectors^15,20^.

As previously noted, the mere elimination of global-scale motion energy (via luminance cancellation or polarity randomization) proved ineffective at eliminating the impression of transparent, global motion in opposite directions. However, reduction of the crests’ contrast did make them harder to see (Supplementary Movie 5). In this case, the retrograde motion of the troughs was perceptually dominant, which we interpret to imply that the opposite directions of crest and trough motions are processed locally by separate local motion mechanisms, which are then each integrated separately around the circle to provide opposing transparent motion percepts. (In terms of the conventional understanding of motion processing, the local motion processing would be by neurons in the cortical motion area hMT, while the global integration would be by cortical optic flow area hMST.) One manipulation that did succeed in eliminating the impression of retrograde motion was the cancellation of the local motion from the troughs (Supplementary Movie 6). The propagation of high-density crests in these latter movies was just perceptible.

The perceptual results were extended by testing for a motion aftereffect generated by longitudinal wave motion. In fact, motion aftereffects are essentially non-existent for the uncompensated longitudinal wave motion (Supplementary Movie 10B), even though strong motion aftereffects are seen for rigidly moving textures of the same format (Supplementary Movie 10A). Given our contention that the impression of transparent global motions arises from large-scale mechanisms that integrate local motion information from individual dots, this negative result may not seem surprising. After all, the inability of other non-Fourier motion stimuli to elicit an aftereffect in static test stimuli has been well established^21–23^. That being said, it is important to remember that black-dot longitudinal waves would indeed be expected to stimulate motion-energy detectors sensitive to the difference between the average luminances of crest and trough. It is therefore surprising to find that the basic longitudinal waves elicit no motion aftereffect, although it can emerge when the contrasts of the components in the two opposing directions are unbalanced. We interpret this result to imply that there are, in fact, aftereffects of the local motion processing units for the opposing directions of the crests and troughs, but that these local aftereffects cancel to produce no net global aftereffect when equated, and that the trough motion aftereffect can be revealed by the manipulation of reducing the contrast of the crest component.

## Supplementary Information

Supplementary Movie 1.

Supplementary Movie 2.

Supplementary Movie 3.

Supplementary Movie 4.

Supplementary Movie 5.

Supplementary Movie 6.

Supplementary Movie 7.

Supplementary Movie 8.

Supplementary Movie 9.

Supplementary Movie 10.

Supplementary Movie Legends

## Data Availability

The code for each movie can be downloaded from the website http://www.staff.city.ac.uk/~solomon/LongitudinalWaves.zip.
