# Supplementary figures and images for: Visual perception of longitudinal waves: theory and observations
Source: Sci Rep. 2026 Mar 23;16:11392. doi: 10.1038/s41598-026-36204-y (PMC13057469; doi:10.1038/s41598-026-36204-y)

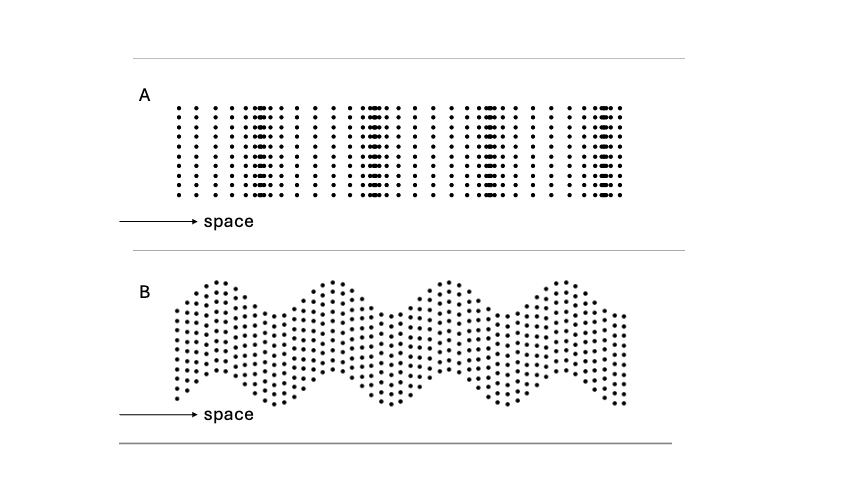

Supplement: Supplementary file 1 — Supplementary Movie 1. [file 41598_2026_36204_MOESM1_ESM.gif]

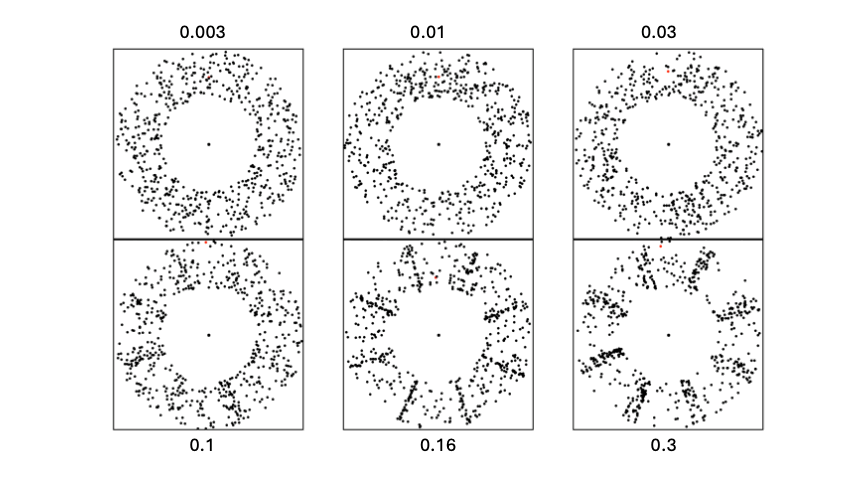

Supplement: Supplementary file 2 — Supplementary Movie 2. [file 41598_2026_36204_MOESM2_ESM.gif]

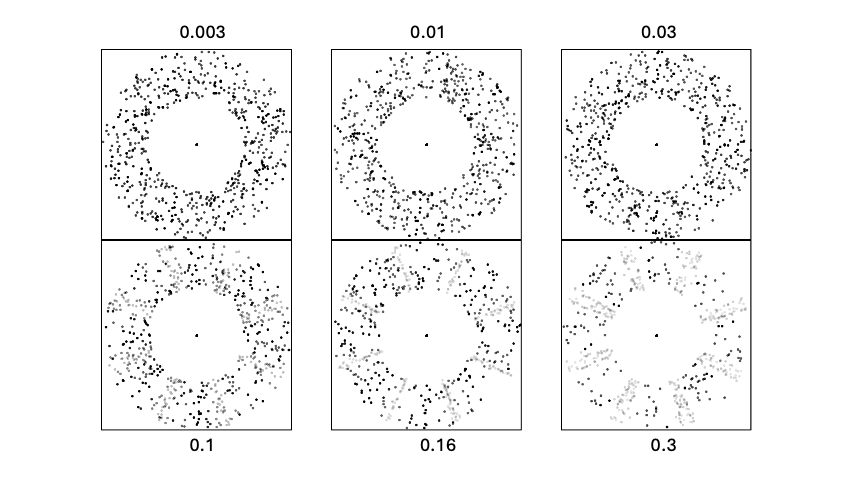

Supplement: Supplementary file 3 — Supplementary Movie 3. [file 41598_2026_36204_MOESM3_ESM.gif]

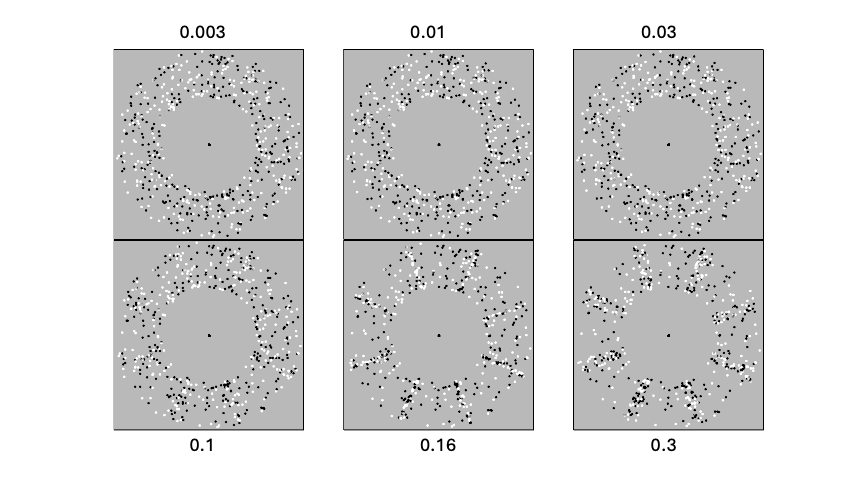

Supplement: Supplementary file 4 — Supplementary Movie 4. [file 41598_2026_36204_MOESM4_ESM.gif]

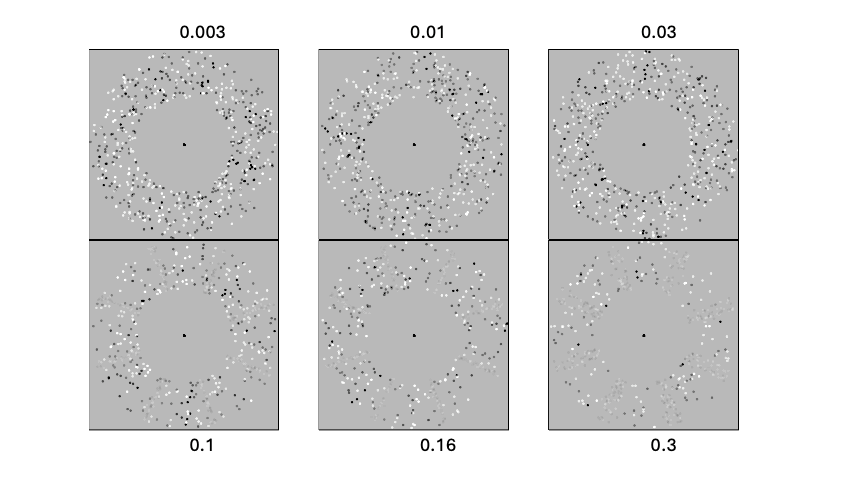

Supplement: Supplementary file 5 — Supplementary Movie 5. [file 41598_2026_36204_MOESM5_ESM.gif]

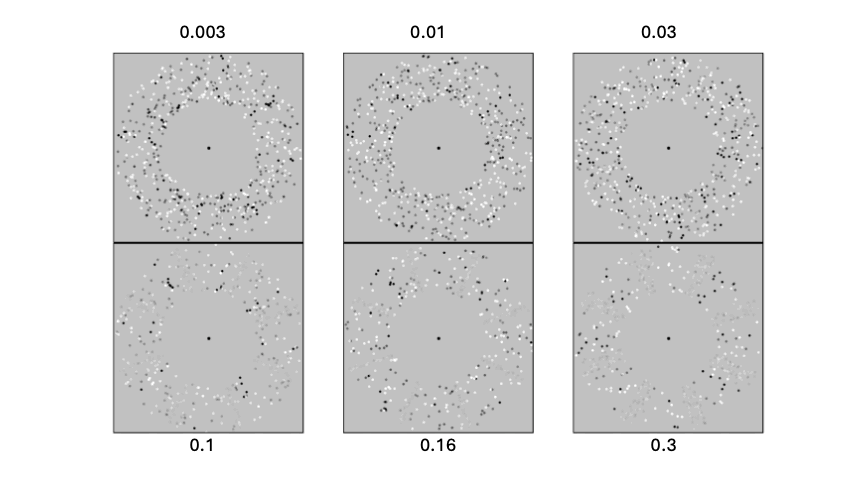

Supplement: Supplementary file 6 — Supplementary Movie 6. [file 41598_2026_36204_MOESM6_ESM.gif]

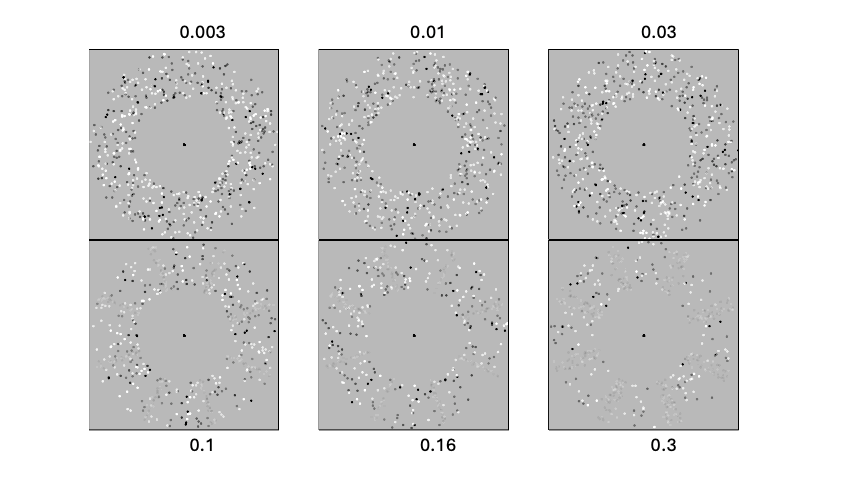

Supplement: Supplementary file 7 — Supplementary Movie 7. [file 41598_2026_36204_MOESM7_ESM.gif]

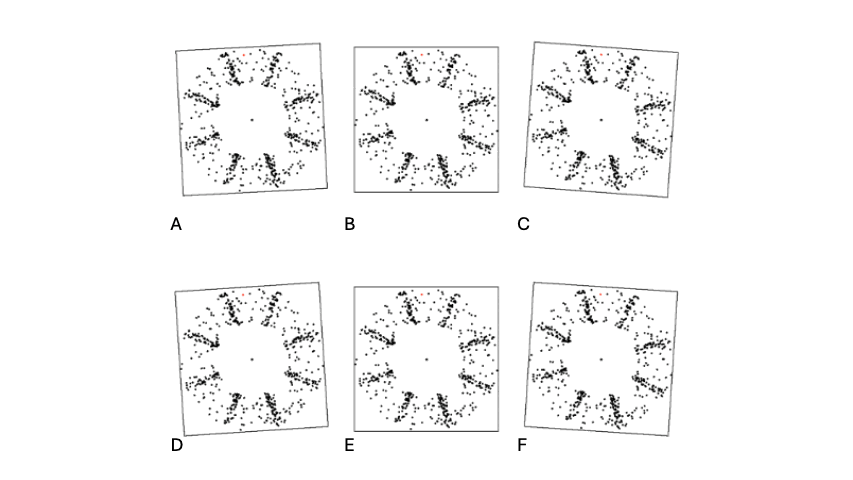

Supplement: Supplementary file 8 — Supplementary Movie 8. [file 41598_2026_36204_MOESM8_ESM.gif]

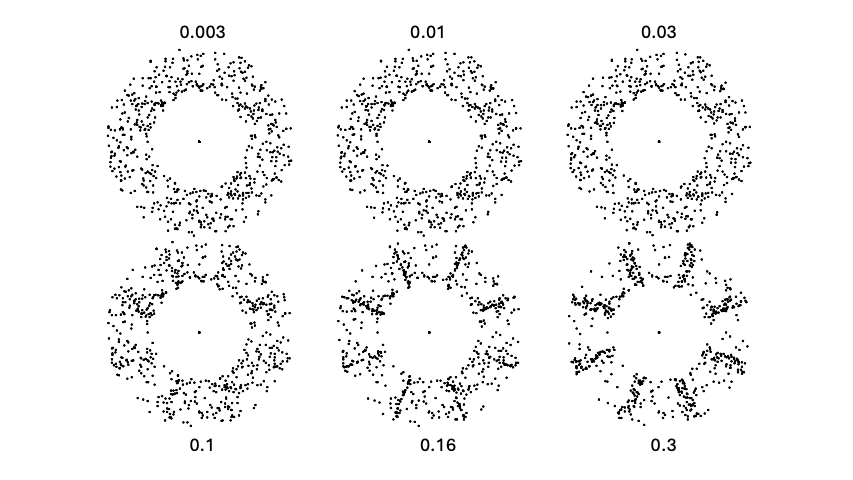

Supplement: Supplementary file 9 — Supplementary Movie 9. [file 41598_2026_36204_MOESM9_ESM.gif]

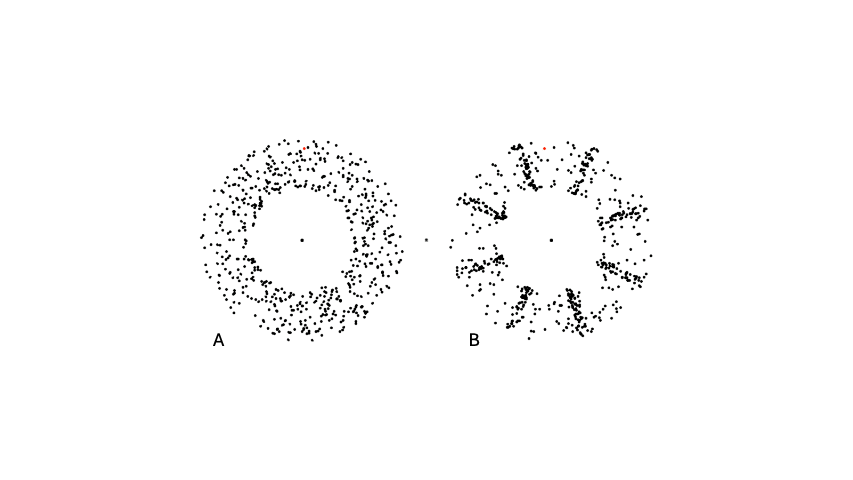

Supplement: Supplementary file 10 — Supplementary Movie 10. [file 41598_2026_36204_MOESM10_ESM.gif]
