# Supplementary material for: Visual perception of longitudinal waves: theory and observations
Source: Sci Rep. 2026 Mar 23;16:11392. doi: 10.1038/s41598-026-36204-y (PMC13057469; doi:10.1038/s41598-026-36204-y)
Supplement: Supplementary file 11 — Supplementary Movie Legends [file 41598_2026_36204_MOESM11_ESM.docx]

Movie 1. Dynamic illustrations of (A) longitudinal and (B) transverse waves, propagating rightward through a grid of dots. (For illustrative purposes, equilibrium positions were equally separated. In all subsequent movies, equilibrium positions were selected at random from a uniform distribution.)

Movie 2. Dynamic illustrations of longitudinal waves, propagating clockwise through annuli of randomly placed dots. The annular format is designed to allow fixation at the center of each annulus to eliminate tracking eye movements. The angle of each dot oscillates sinusoidally in place with an amplitude ranging from $0.003\lambda$ (top left) to $a=0.3\lambda$ (bottom right), as labeled. The red dot near the top is designed to aid verification that each dot is oscillation in place. The motion conditions match those diagrammed in Fig. 2. In all panels, the phase propagation speed is 42 polar deg/s. Wave motion is visible for oscillation amplitudes above ~$0.01\lambda$.

Movie 3. Luminance-balanced version of Movie 2, in which dot luminance varies in proportion to dot density. Wave motion is easily visible from about$0.03\lambda$.

Movie 4. Drift-balanced version of Movie 2, in which dot polarity has been randomized. Wave motion is easily visible from about$0.03\lambda$.

Movie 5. Contrast-balanced version of Movie 2, in which dot contrast varies inversely with dot density. Wave motion is easily visible for $0.03\lambda-0.1\lambda$ but not for higher amplitudes.

Movie 6. Contrast-balanced density waves as in Movie 5 but modified to eliminate local dot motions by randomizing the radial position of each dot. Propagation of individual crests can be seen with effort at high amplitudes.

Movie 7. Flickering version of Movie 5, in which dot polarity is randomly reassigned on each frame.

Movie 8. Different levels of rigid rotational motion added to each dot of high-amplitude ($0.25\lambda$) longitudinal waves. Motion of the frames is keyed to the added motion to give a clear indication of its angular velocity. A: With matching reverse added motion (clockwise velocity –$1.05c$). B,E: No added motion. C: Matching forward added motion (clockwise velocity +$1.50c$). D,F: Equal but non-matching intermediate speed (clockwise velocity +$1.30c$). Note that the crests appear stationary in panel A and the trough regions appear stationary in panel C. In panels D and F, neither the crests nor the troughs appear stationary (both appear to rotate counterclockwise) but the frames rotate at the same speed. Panels 8D and F illustrate the same high-amplitude longitudinal wave with equal but opposite rigid velocities of +1.3*c*. This intermediate velocity proves too fast to cancel the crests’ motion (they appear to rotate counterclockwise in 8D) and too slow to cancel the troughs’ (they appear to rotate counterclockwise in 8F). Quantitative cancellation speeds for lower-velocity waves are plotted in Fig. 4.

Movie 9 contains the same longitudinal waves shown in Movie 2, with the wave’s actual propagation velocity subtracted from each dot. This is approximately the correct speed for cancelling the apparent propagation of the crests only when the oscillation amplitude $a\approx0.2\lambda$. For larger amplitudes, it is insufficient, and the crests appear to rotate clockwise. For smaller amplitudes, it is overkill, and any discernible crests appear to rotate counterclockwise.

Movie 9. Identical levels of counterclockwise motion with propagation speed $c$ added to Movie 2 modified by adding identical levels of counterclockwise motion at its phase propagation speed of $c$ = 42 deg/s. This is too slow to cancel the apparent clockwise propagation when $a=0.3\lambda$ (bottom right) but too fast to cancel apparent propagation when $a<0.2\lambda$.

Movie 10. Motion aftereffect and lack thereof. A 2.13-s static test period follows 10.67 s of motion adaptation with a uniform texture rotating at 6.3 deg/s (panel A) and a high-amplitude ($0.25\lambda$) longitudinal wave propagating at 42 deg/s (panel B). The uniform angular velocity in panel A is set to the sum of the rigid velocities required to cancel the two directions of transparent motion in panel B (see Movies 8A–C). A strong motion aftereffect is seen during the static test period with centered adaptation in panel A, but not for the same in panel B.
